# Single-cell transcriptomic analysis of the human vascular atlas provides new insights into vasorelaxation redundancy and heterogeneity

**DOI:** 10.3389/fcvm.2025.1634645

**Published:** 2025-08-13

**Authors:** Elisa Avolio, Sarah F. Pearce, David Wambeke, Paolo Madeddu

**Affiliations:** ^1^ Experimental Cardiovascular Medicine, University of Bristol, Bristol, United Kingdom; ^2^ DiaMedica Therapeutics, Minnetonka, MN, United States

**Keywords:** endothelial nitric oxide synthase (eNOS), bradykinin receptors (b1R/b2R), soluble guanylate cyclase (sGC), guanosine triphosphate (GTP), protein kinase g (PKG), prostaglandin H2 (PGH2), prostaglandin H synthase (PGHS)

## Abstract

**Background:**

Endothelial cells (ECs) induce vascular smooth muscle cells (VSMCs) relaxation via nitric oxide (NO), prostacyclin (PGI₂) and hyperpolarizing factors. Recent whole-genomic, single-cell transcriptomic analysis of human vascular cells has revealed angiotypic heterogeneity. However, it remains unknown whether vasorelaxant mediators reiterate this pattern.

**Working hypothesis:**

The expression of “sentinel” gene transcripts provides a first insight into angiotypic and organotypic heterogeneity of vasorelaxation.

**Methods:**

The expression of NO- and PGI_2_-generating enzymes and potassium channels was evaluated by analyzing single-cell RNA-sequencing data derived from the Human Vascular Cell Atlas. The data were transformed into a Seurat object (ShinyCell) and processed using the RStudio software. The results were visualized through uniform manifold approximation (UMAP) and projection representations of single-cell profiles.

**Results:**

NO synthase (*NOS3*) expression differed across EC subpopulations, with the highest enrichment in spleen littoral ECs, followed by venous, arterial, and capillary ECs. PGI_2_ synthase (*PTGIS*) demonstrated the highest frequency in arterial and venous ECs and VSMCs. At the same time, it was low in capillary, littoral, and lymphatic ECs and pericytes. The PGI_2_ receptor gene (*PTGIR*) was expressed in vascular mural cells. A marked angiotypic heterogeneity was noted regarding potassium channels. Overall, the gene transcripts mentioned above were rarely co-expressed. Comparing two cohorts aged 20 to 49 and 50 to 80 revealed *NOS3* expression was less frequent in venous and littoral ECs of older individuals. In contrast, arterial and capillary ECs were modestly affected by age. *PTGIS* frequency was elevated with aging in VSMCs and, to a lesser extent, in venous and arterial ECs. The *KCNMA1* gene, which encodes the big potassium channel alpha subunit 1, was almost doubled in the VSMCs from the older group. Finally, organotypic differences were identified in vascular cells derived from the coronary arteries, brain, and uterus.

**Conclusion:**

This initial report indicates a striking heterogeneity in the expression of genes encoding vasorelaxant pathways in human vascular cells, with age exerting angiotypic influences. Reiteration on a larger number of cells and genes, as well as validation of data using post-transcriptional methods, is warranted to firmly confirm the hypothesis about redundant heterogeneity of vasorelaxant mechanisms.

## Graphical Abstract

**Figure:**
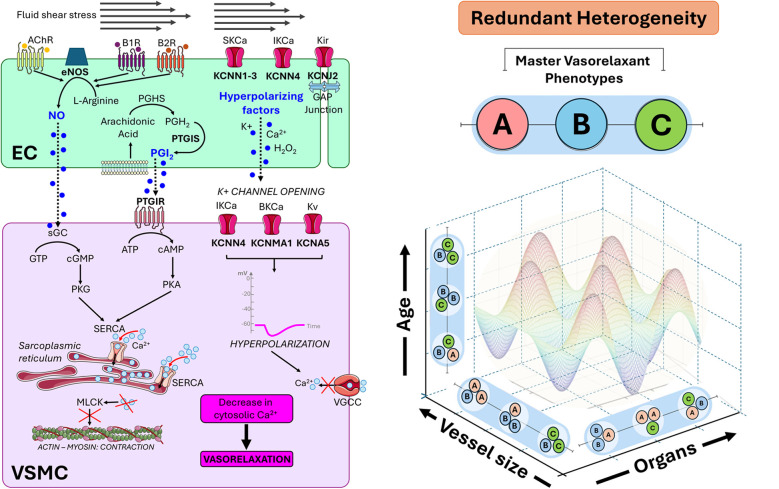
On the **left-hand side**, the representation of the signalling that originates from endothelial cells (EC) and propagates to vascular smooth muscle cells (VSMC), causing a reduction in cytosolic calcium levels in the latter and subsequent relaxation. Three endothelium-derived relaxing factors—nitric oxide (NO), prostacyclin (PGI_2_), and hyperpolarizing factors (all indicated in blue)—signal in neighbouring VSMCs through distinct mechanisms. NO and PGI_2_ signal through the second messengers cyclic guanosine monophosphate (cGMP) and cyclic adenosine monophosphate (cAMP), respectively, enhancing calcium (Ca^2+^) uptake into the sarcoplasmic reticulum *via* sarco/endoplasmic reticulum Ca^2+^-ATPases (SERCAs). Hyperpolarizing factors promote the opening of potassium (K^+^) channels, leading to membrane hyperpolarization and the subsequent closure of voltage-gated calcium channels (VGCCs). These mechanisms converge to lower cytosolic Ca^2+^ levels, diminishing myosin light chain kinase (MLCK) activation, and ultimately promoting vasorelaxation. On the **right-hand side**, the concept of redundant heterogeneity is introduced. The multiplicity of vasorelaxant mechanisms ensures a first-tier redundancy, which is heterogeneously distributed among vascular cells according to angiotypic (vessel size) and organotypic (different organs) blueprints. Redundant heterogeneity confers the vascular system with peculiar flexibility and resilience to stress, disease, and aging. The coloured harmonic function image exemplifies how the dynamic interaction between vasorelaxant phenotypes and environmental factors results in functional adaptations, with hills and valleys representing increased or reduced vasorelaxation**.** AchR, acetylcholine receptor; eNOS, endothelial nitric oxide synthase; B1R/B2R, bradykinin receptors; sGC, soluble guanylate cyclase; GTP, guanosine triphosphate; PKG, protein kinase G; PGH_2_, Prostaglandin H2; PGHS, prostaglandin H synthase; PTGIS, PGI_2_ synthase; PTGIR, PGI_2_ receptor; ATP, adenosine triphosphate; PKA, protein kinase A; H_2_O_2_, hydrogen peroxide; KCa, potassium calcium-activated channel; Kv, potassium voltage-gated channel; Kir, potassium inward-rectifier channel; SKCa, small conductance calcium-activated potassium channel; IKCa, intermediate-conductance calcium-activated potassium channel; BKCa, big-conductance potassium channel; KCNN1-4, potassium calcium-activated channel subfamily N member 1-4; KCNJ2, inward rectifier K+ channel protein Kir2.1; KCNMA1, calcium-activated potassium channel subunit alpha-1; KCNA5, potassium voltage-gated channel subfamily A member 5. Images from Smart Servier Medical Art: “Receptor green”, “Receptor orange”, “Receptor purple”, Receptor”, “Ion red receptors channel”, “Pink channel”, “Beige receptors channel”, “Tissue junction overview”, “Cell membrane”, “Endoplasmic reticulum” and “Actin”, licensed under CC BY 4.0.

## Introduction

The cardiovascular system comprises the heart and a network of large elastic arteries, resistance arteries, arterioles, and terminal capillaries, connected to the venous return. Each arterial segment consists of three concentric layers: (i) the intima, lined with endothelial cells (ECs) that directly interface with the vessel lumen; (ii) the tunica media, which is made up of at least one layer of vascular smooth muscle cells (VSMCs); and (iii) the adventitia, which contains mesenchymal cells, pericytes, immune cells, and nerve terminals. In larger arteries, this outer layer includes a specialized vascular supply system termed “vasa vasorum”. In contrast to arteries and arterioles, capillaries do not possess a VSMC sheet. They are composed of continuous tubes of ECs, covered by a basement membrane and pericytes. These perivascular cells physically and functionally interact with the capillary endothelium through adhesion plaques, gap junctions, and peg-and-socket structures.

Compared to their diameter, arterioles contain a higher proportion of VSMCs and less elastin than large arteries, which accounts for their role as major regulators of vascular resistance and regional blood flow. The tiny capillaries deliver nutrients and oxygen to cells and remove waste products, while also contributing to blood flow modulation through the pericytes' ability to contract and relax. The endothelium acts as a master regulator of vasorelaxation, sensing and transducing chemical stimuli, such as acetylcholine (Ach) and bradykinin (BK), or mechanical forces, such as fluid shear stress from within the artery, into intracellular signalling. The process involves the secretion of diffusible transmitters by ECs, specifically nitric oxide (NO), prostacyclin (PGI_2_), and the so-called hyperpolarizing factors, which interact to reduce cytosolic Ca^2+^ levels in VSMCs, allowing relaxation (1). Intercellular gap junctions facilitate bidirectional relaxation currents between small arterioles and upstream arteries, greatly enhancing tissue perfusion (2, 3).

The three endothelium-derived relaxing factors trigger intracellular Ca^2+^ changes using distinct second messengers.

(i) The endothelium-specific NO synthase (eNOS) is responsible for approximately 90% of NO generation from the L-arginine substrate. The signalling from NO involves the activation of soluble guanylate cyclase (sGC), which catalyzes the conversion of guanosine triphosphate (GTP) into inorganic pyrophosphate and the secondary messenger cyclic guanosine monophosphate (cGMP). CGMP activates protein kinase G, which reduces the cytosolic Ca^2+^ levels by promoting its uptake by sarcoplasmic reticulum calcium-ATPases (SERCA) (4, 5).
(ii) PGI_2_ is a terminal product resulting from the sequential metabolism of arachidonic acid by cyclooxygenase (COX) and prostacyclin synthase (PGIS). The receptor for PGI_2_, known as IP, is present on various cell types, and signaling through this receptor demonstrates extensive physiological actions, particularly for its potent vasodilatory effects through smooth muscle relaxation and inhibition of platelet aggregation (6). By binding the IP receptor, encoded by the *PTGIR* gene and highly expressed in the platelets, lung, heart, kidney, and throughout the vasculature, PGI_2_ triggers physiologic responses mediated by the intracellular accumulation of cyclic adenosine monophosphate (cAMP). CAMP activates protein kinase A (PKA), promoting sarcoplasmic reticulum Ca^2+^ uptake and inhibiting MLCK *via* phosphorylation (7).
(iii) Hyperpolarizing signalling is a prevalent relaxation mechanism at the level of small arterioles. It functions independently of NO and PGI_2_, becoming dominant when the two other vasorelaxant pathways are suppressed pharmacologically or downregulated due to endothelial dysfunction (8–10). Hyperpolarization initiates in ECs and propagates to VSMCs *via* chemical signals, such as potassium (K^+^), lipid mediators, and hydrogen peroxide, as well as through electrical coupling through myoendothelial gap junctions (10). A crucial step in the hyperpolarization cascade is represented by the opening of K+ channels. The most prominent family of K+ channels are activated by membrane depolarization, with other families consisting of channels that are either activated by a rise in intracellular calcium ions or are constitutively active. A standardized nomenclature for K+ channels has been proposed by the NC-IUPHAR Subcommittees, which has placed cloned channels into groups based on gene family and structure of channels that exhibit 6, 4 or 2 transmembrane domains (11). In the present study, we focused on the following channels: Ca^2+^-activated K+ (KCa), voltage-gated K+ (Kv), and inward-rectifier K+ (Kir) channels. (i) *KCa channels* comprise three subtypes: the small-conductance channels (SKCa), intermediate-conductance channels (IKCa), and big-conductance channels (BKCa) (12). (ii) *KV channels* are activated at more negative membrane voltages than KCa in VSMCs of small arterioles, thus conspicuously contributing to vascular tone control (13). (iii) *Kir channels*, considered the most prominent channels in ECs but also expressed by capillary pericytes, are activated by laminar flow, G protein-coupled receptor (GPCR) agonists, and K^+^ efflux from neighbouring cells (14). Kir channels enable a large K^+^ influx but little outward current outflow under physiological conditions (14). This causes additional K^+^ efflux and an enhancement of hyperpolarization, which can be eventually transmitted through the endothelial layer to dilate upstream feeding arteries (15).

Over the past twenty years, the concept of vascular cell heterogeneity has revolutionised the field of vascular biology (16). Vascular cells exhibit differences among various organs and tissues, as indicated by variations in overall morphology, intracellular organisation, and gene expression profiles. Furthermore, significant differences in functional characteristics distinguish cells of large vessels from those of the microvasculature, likely due to epigenetic programming, as these differences persist even after isolation and *in vitro* culture (17, 18). The heterogeneities and the redundancy of vasorelaxant processes are essential for providing the necessary flexibility to perform various physiological duties. Classical research using isolated vascular models and pharmacological inhibitors has helped decipher regional variations in the expression and function of vasorelaxation-related mechanisms. Analyses using single-cell RNA transcriptomics have demonstrated angiotypic and organotypic parallels and diversities that could not be discriminated with classical pharmacological approaches (19, 20). It would be of paramount importance to apply the same approach to decipher vascular vasorelaxant transcriptomic profiles. Filling this knowledge gap will yield substantial clinical and therapeutic consequences. For instance, it may facilitate the development of novel pharmaceuticals that specifically target particular artery beds and organs, thereby reducing off-target effects.

This study conducted a qualitative transcriptomic analysis of key vasodilator pathways in vascular cell subtypes at both the organismal and regional levels, aiming to inform and establish a theoretical basis for future quantitative research. Our working hypothesis was that the expression of “sentinel” gene transcripts would provide a first insight into the angiotypic and organotypic heterogeneity of vasorelaxation. We extracted and analyzed single-cell RNA sequencing data on enzymes, receptors, and channels involved in vasorelaxant pathways from a publicly available Human Vascular Atlas (20). This database provides detailed molecular profiles of endothelial and mural cells, offering a framework for investigating physiological adaptations across various vascular beds in different organs and tissues (20). Results indicate previously unforeseen disparities between vascular cells from arterioles and capillaries or different organs, with further changes being imposed by aging.

## Methods

The Human Vascular Atlas is a comprehensive collection of vascular cells from 19 organs and tissues. It integrates arterial, capillary, venous, spleen pulp (littoral), and lymphatic ECs, pericytes, and VSMCs, yielding approximately 67,000 cells from 62 male and female donors. As described in detail previously (20), datasets, other than Tabula Sapiens data, were mapped using CellRanger (version 3.0) and STARsolo (version 2.7.3a) to the human genome (GRCh38 version 3.0.0), with ambient RNA removed using CellBender (version 0.2.0). Pre-processed Tabula Sapiens data were used. Python (version 3), AnnData (versions 0.8.0 and 0.9.1) Pandas (versions 1.5.2 and 1.5.3), NumPy (versions 1.21.6 and 1.23.5), SciPy (versions 1.8.0 and 1.9.3), Matplotlib (version 3.5.2), Seaborn (version 0.12.2) and Scanpy (versions 1.8.2 and 1.9.1) were used for QC and downstream processing. Doublets were identified utilizing Scrublet (version 0.2.3), with cells exhibiting a Scrublet score ≥ 0.4 and an adjusted Benjamini–Hochberg-corrected *P* ≤ 0.7 classified as doublets and subsequently excluded. Cells of inferior quality were excluded based on the following criteria: minimum read count = 500, minimum gene count = 300, mitochondrial gene percentage ≤ 0.4, ribosomal gene percentage ≤ 0.3. The data were subsequently standardized to 104 and log-transformed [ln(x + 1)] utilizing the SCANPY workflow115. Untransformed reads were preserved in the layer designated as “counts”.

We employed the ShinyCell R package (v2.1.0) to interrogate the associated single-cell RNA-sequencing data to visualize cell metadata and gene expression. The fully interactive Shiny application enables the generation of low-dimensional uniform manifold approximation and projection (UMAP) embeddings, as well as the simultaneous investigation of expression patterns of multiple genes through bubble plots and heatmaps. Briefly, the “Vascular Cell Atlas” dataset (.h5ad, AnnData file) was downloaded in.h5ad format (AnnData file) from https://www.vascularcellatlas.org/ and converted into a Seurat object using the MuDataSeurat package (v 0.0.0.9000). Finally, using the ShinyCell R package, a ShinyCell application was generated from the Seurat object and run via RStudio for data analysis. ShinyCell generation was performed using R (version 4.4.3) and Seurat (version 5.2.1).

Considering the exploratory nature of our study, along with the limited number of cells and donors, we adopted a descriptive approach to reporting the results, without performing quantitative statistical comparisons.

## Results

### Expression of vascular typical genes

As reported in Supplementary Table S1, ECs exhibited the expected high-level expression of *CDH5*, *VWF*, *PECAM1*, *EGFL7*, and *VWF*. These genes were also co-expressed by single cells, as in the case of the *PECAM1*—*EGFL7* duo, showing a double-positive signal in 73% of ECs from capillaries and arterioles. At the same time, mural cells could be classified as VSMCs and pericytes based on the expression of classical markers, including *PDGFRB*, *NOTCH3*, *ACTA2*, *MYH11*, and *RGS5*.

### Angiotypic differences in the expression of vasorelaxation-related genes

**Figure 1:**
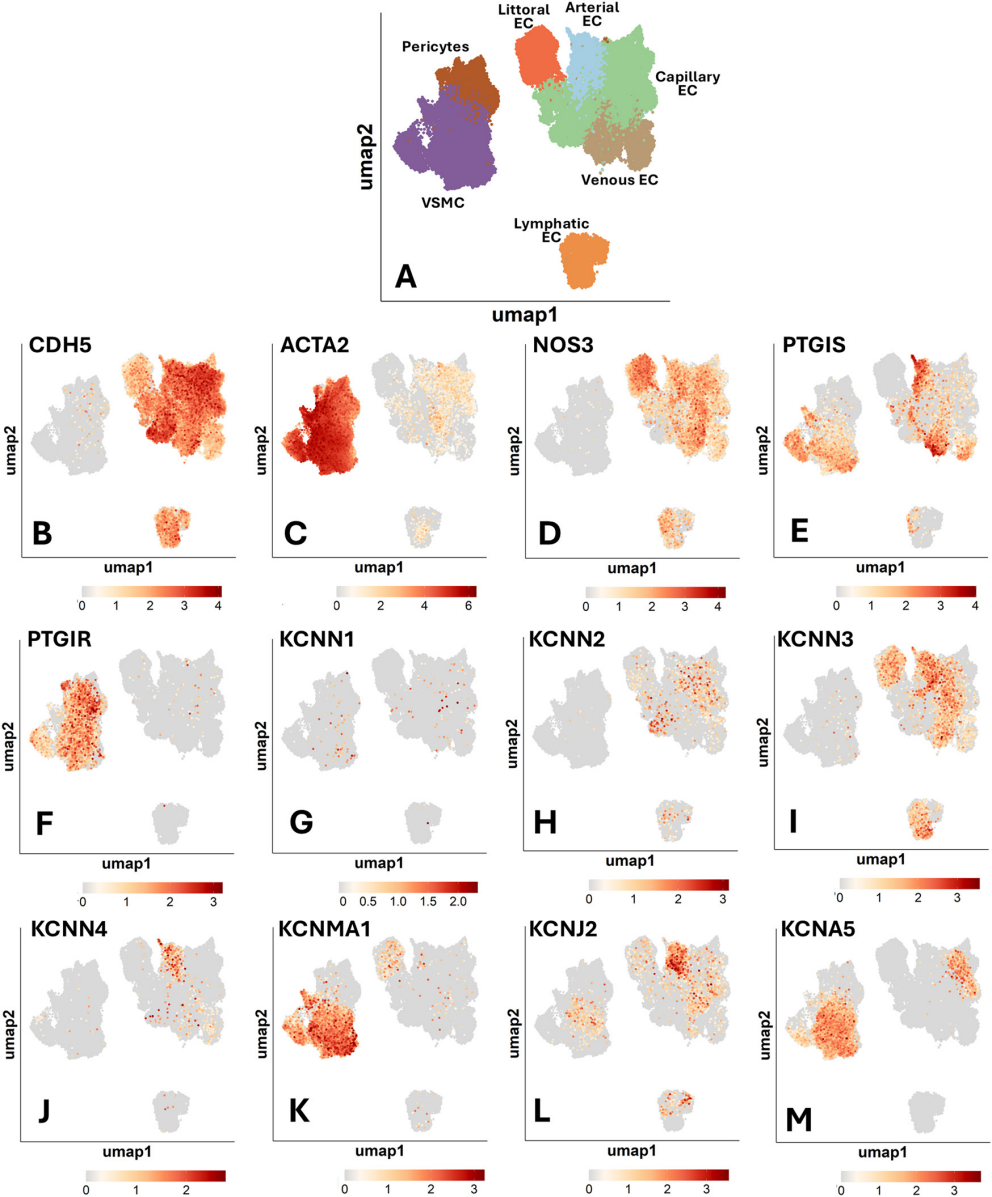
Uniform manifold approximation and projection (UMAP) representations of single-cell profiles and gene expression, side by side. The red scale indicates the level of gene expression. **(A)** UMAP representations of single-cell profiles. **(B)** CDH5; **(C)** ACTA2; **(D)** NOS3; **(E)** PTGIS; **(F)** PTGIR; **(G)** KCNN1; **(H)** KCNN2; **(I)** KCNN3; **(J)** KCNN4; **(K)** KCNMA1; **(L)** KCNJ2; **(M)** KCNA5.

We next analyzed a detailed annotation of organismal vascular cells. Figure 1A illustrates UMAP representations of single-cell profiles. Figures 1B,C visualizes the expression of endothelial and mural typical genes: *CDH5*, which encodes VE-cadherin, and *ACTA2*, which encodes alpha-smooth muscle actin. The following panels (Figures 1D–M) represent the UMAP superimposed with expression profiles of genes of interest, with cell type-specific percentage expression reported in Supplementary Table S2.

### Nitric oxide synthase

The expression of eNOS was reported to be highly restricted to the endothelium of medium to large arterial blood vessels (21). We observed a marked variation in the abundance of *NOS3*-positive ECs across the different vascular territories (Figure 1D). When considering the frequency of positive cells, a term used hereon to indicate the proportion of cells with detected expression of a given gene, we found that littoral ECs, spleen sinusoids-lining cells specialized in scavenging senescent blood cells, were the most copious in *NOS3* transcripts (62% positive cells). They were followed by venous ECs (33%), arterial ECs (27%), and capillary and lymphatic ECs (both 16%).

### Prostacyclin and cognate receptor

PTGIS (Prostacyclin/Prostaglandin I2 synthase) and PTGIR (Prostacyclin/ prostaglandin I2 receptor) are reportedly localized in both ECs and VSMCs (22, 23). As shown in Figure 1E, single-cell analysis revealed a distinct expression pattern for *PTGIS*, which exhibited the highest frequency in arterial and venous ECs (25% and 27%, respectively). In contrast, *PTGIS* was low-abundant in capillary (3%), lymphatic (2%) and littoral ECs (<1%). Among mural cells, VSMCs expressed *PTGIS* (22%), whereas pericytes were negative. On the other hand, *PTGIR* expression was enriched explicitly in mural cells, with similar frequencies in pericytes and VSMCs (17% and 18%, respectively) (Figure 1F). Again, these data support an angiotypic heterogeneity with a distinct pattern compared with *NOS3*.

### Potassium channels

Within the category of potassium channels (Figures 1G–M), *KCNN1* (SKCa1) and *KCNN2* (SKCa2) demonstrated low overall expression levels. Regarding the other expressed channels, *KCNN3* (SKCa3) was variably expressed among the EC subtypes, with the highest frequency in arterial ECs (23%), followed by littoral ECs (17%), lymphatic ECs (15%), venous ECs (12%), and capillary ECs (5%). In contrast, it was absent in mural cells. Both Kir and Kv channels play a key role in vascular physiology. We found that *KCNN4* (IKCa) and *KCNJ2* (Kir2.1) were mainly expressed by arterial ECs (8% and 19%, respectively). *KCNMA1* (BKCa) was expressed in VSMCs (37%) and was low-expressed in ECs and pericytes. Kv channels are encoded by a large set of KCNx genes. We found that, within this channel family, only *KCNA5* (Kv1.5) was detectable in the Human Vascular Atlas database, being expressed in VSMCs (32%) and to a much lesser extent in capillary ECs (2%). Following a specific request from one reviewer, we have examined the expression of *KCNQ* channels (Kv7), voltage-gated, phosphatidylinositol 4,5-bisphosphate (PIP 2-) modulated K+ channels that play essential roles in regulating the activity of neurons and cardiac myocytes, but also vascular cells. The data showing low expression levels of these channels in the general cell population are reported in Supplementary Table S2. In summary, K+ channels are expressed differently depending on the subtype of vascular cell population.

### Gene co-expression

**Figure 2:**
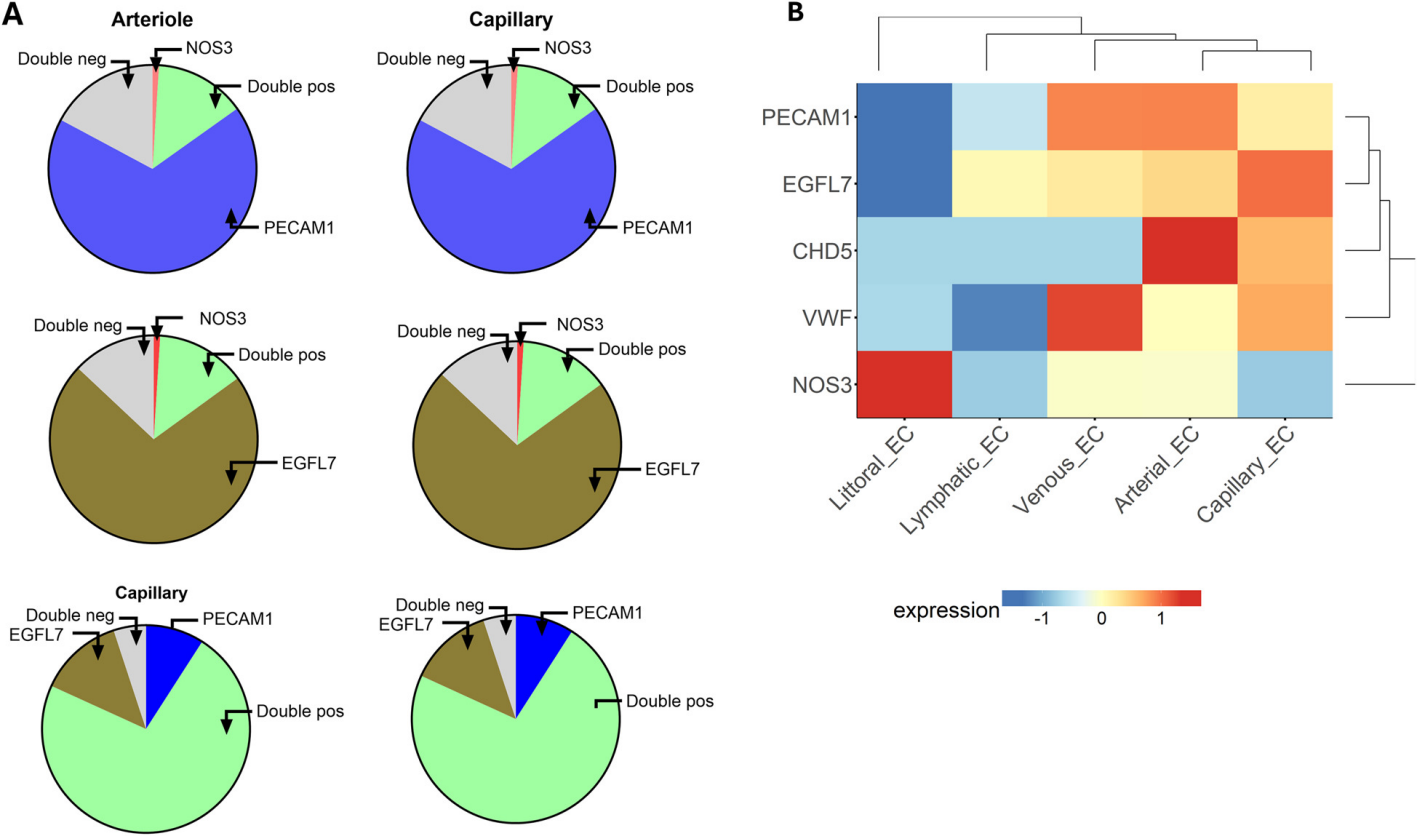
**(A)** The graphic illustrates the proportions of ECs expressing the indicated genes alone or in combination (double positive) or negative for both genes (double negative). Top, Expression of *NOS3* and *PECAM1*; Middle, *NOS3* and *EGFL7*; Bottom, *PECAM1* and EGFL7. **(B)** Heatmap of endothelium-related genes in cells from different vascular compartments.

The expression of eNOS is generally considered typical of endothelial cells. In line with this, *NOS3*-expressing ECs were also positive for canonical endothelial markers, such as *PECAM1* and *EGFL7*. However, as inferred from the percentages mentioned above and illustrated in Figure 2A, a larger proportion of PECAM1- or EGFL7-positive ECs, particularly those from the capillary district, were NOS3-negative. A double negative fraction could also be detected. This data further highlights EC heterogeneity. Figure 2B shows the heatmap of *NOS3* and other endothelium-related genes grouped by categorical cell information.

**Figure 3:**
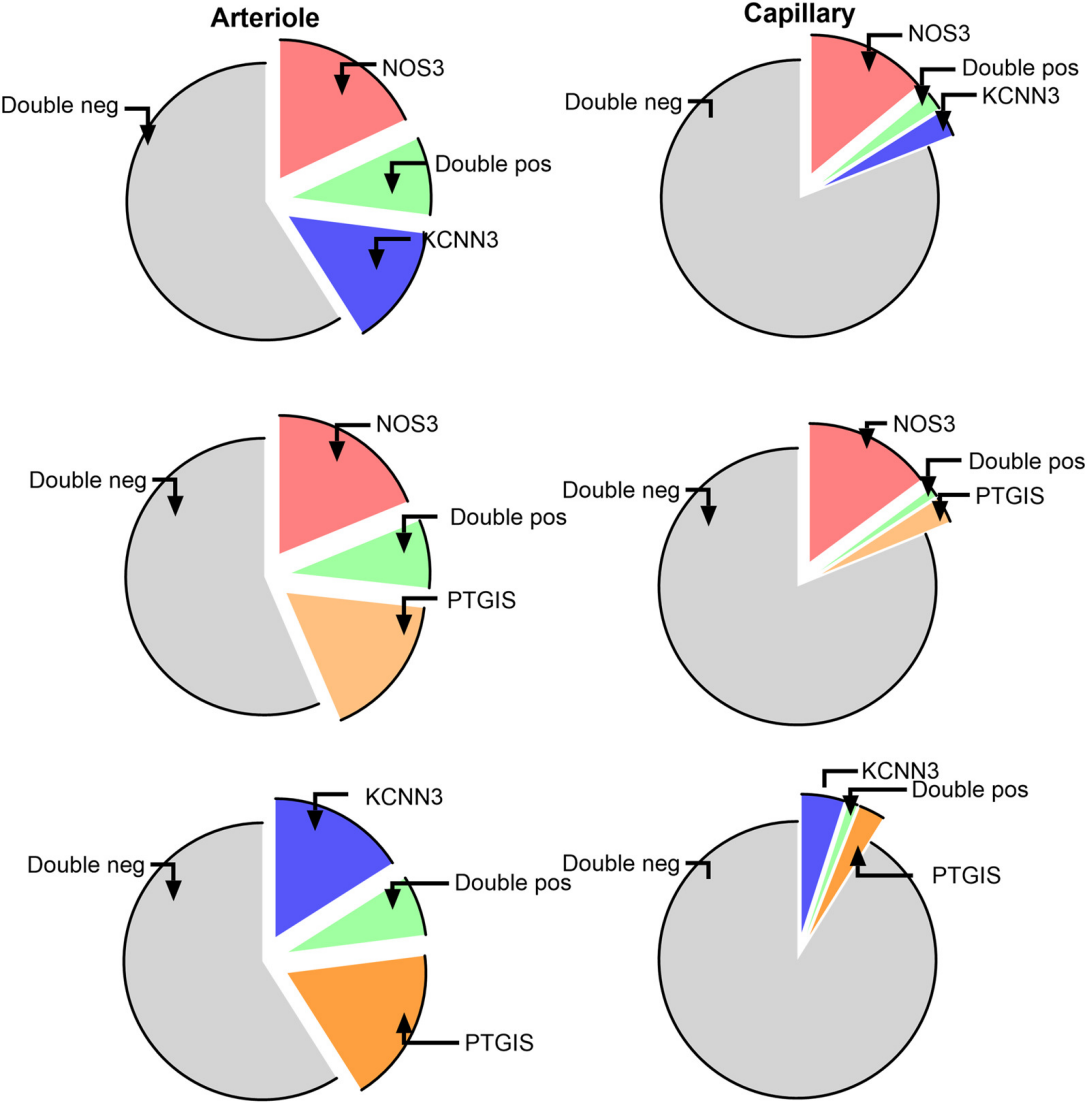
Co-expression of the most represented genes in ECs from arterioles and capillaries. TOP, *NOS3* and *KCNN3*; Middle, *NOS3* and *PTGIS*; Bottom, *KCNN3* and *PTGIS*.

We next considered the co-expression levels of the most represented genes, namely *NOS3*, *PTGIS*, and *KCNN3*, in ECs from arterioles and capillaries. As shown in Figure 3, a fraction between 7% and 9% of arterial ECs were double positive, while more than 50% resulted double negative for any possible combinations. The co-expression dropped to 1% or less when considering capillary ECs, with 81 to 92% being double negative. In conclusion, only a modest fraction of arterial ECs, and an even lower proportion of capillary ECs, displayed detectable combinatory expression for the three considered genes.

### Clustering analysis

**Figure 4:**
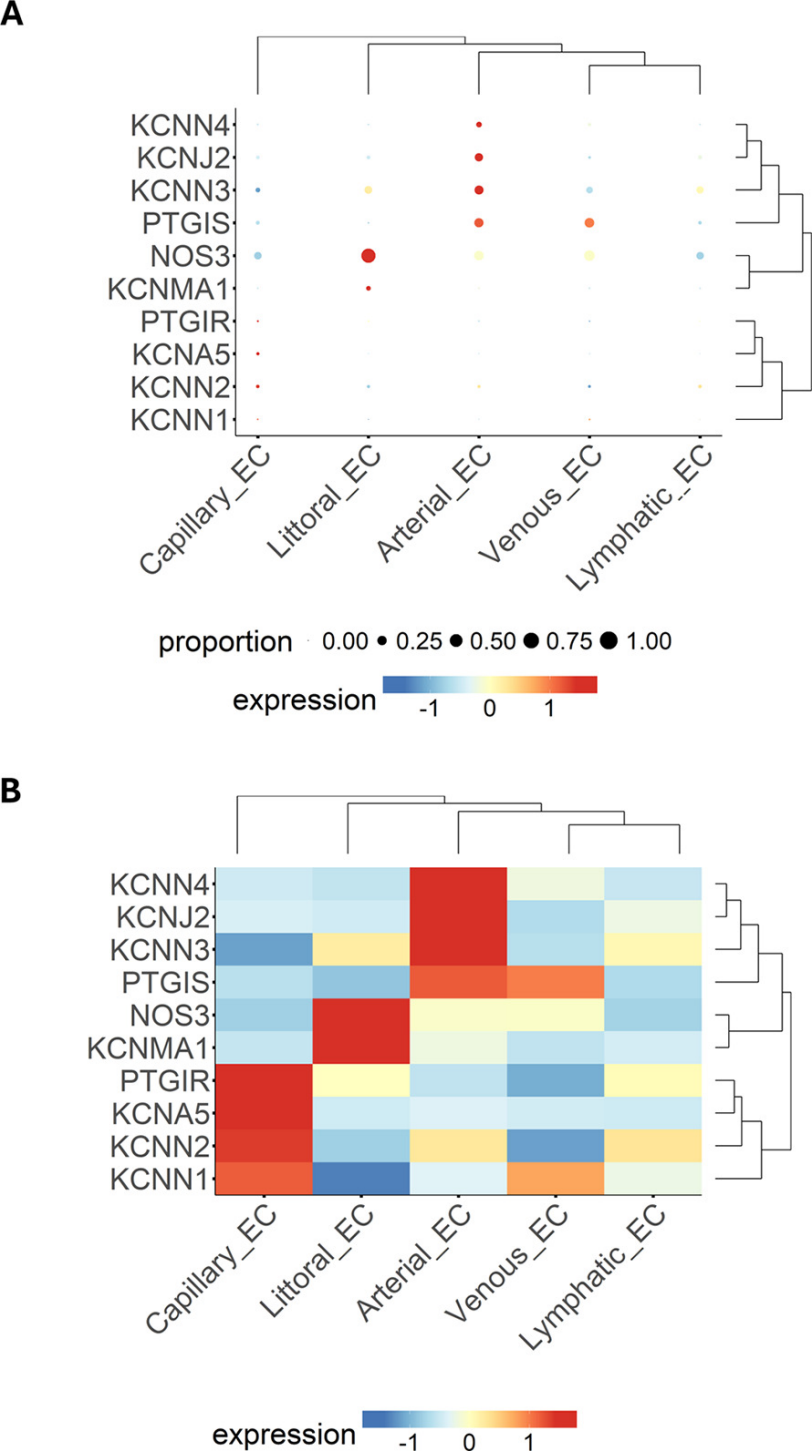
Expression patterns of multiple genes, grouped by categorical cell information, are displayed as either a bubble plot **(A)** or a heatmap **(B)**. Genes and cell populations are grouped into clusters, and dendrograms illustrate their relative distance. Bubble dimensions are proportional to the number of positive cells, while the heatmap colours identify the expression level as indicated by the scale.

Next, we examined the relationship between genes and EC subpopulations, illustrated as a bubble plot (Figure 4A) and a heatmap (Figure 4B), where the colour scale indicates the gene expression level. The dendrogram displays the clustering of samples or genes. Results again highlight the wide heterogeneity of ECs, with the most considerable distance observed between capillary ECs vs. lymphatic ECs.

### Changes in gene expression with ageing

**Figure 5:**
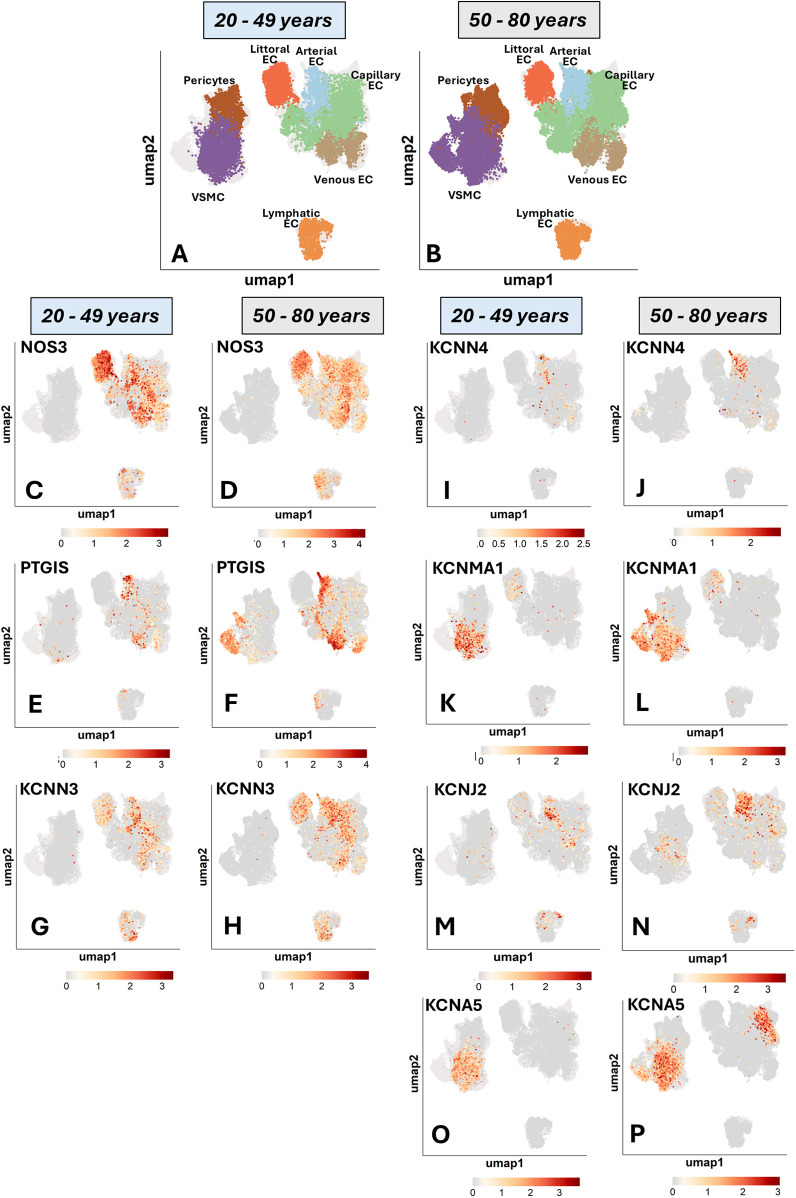
UMAP representations of single-cell profiles and gene expression. The red scale indicates the level of gene expression. (**A,B)**: cell distribution. **(C,D)**: *NOS3*. **(E,F)**: *PTIGS*. **(G,H)**: *KCNN3*. **(I,J)**: *KCNN4*. **(K,L)**: *KCNMA1*. **(M,N)**: *KCNJ2*, and **(O,P)**: *KCNA5*.

Advancing age is associated with structural, molecular, and functional alterations affecting all cellular components of the vasculature (24, 25). In this context, hyperpolarization-dependent vasodilation is believed to be a backup system for decreasing NO and PGI_2_ production. We tested whether older age is associated with transcriptional changes at the single-cell level. To enhance the power of this analysis, we compared two cohorts aged 20 to 49 and 50 to 80 for the most relevantly expressed genes. Figure 5 shows the UMAP representation of gene expression for the two age strata side by side. These data suggest age is a contributor to vascular cell heterogeneity.

**Figure 6:**
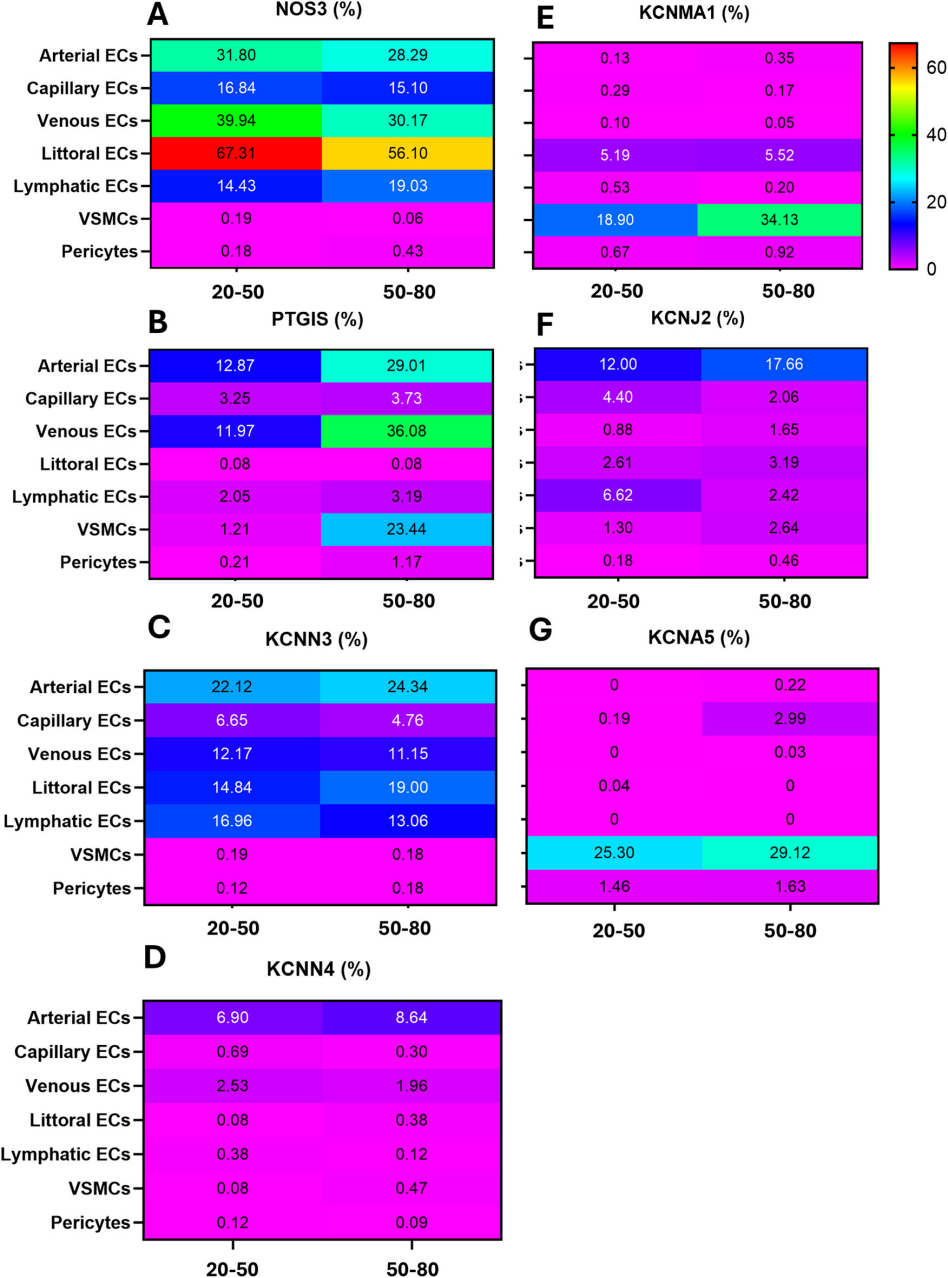
The rainbow heatmap shows the percentage of cells that express a given gene. **(A)** NOS3; **(B)** PTGIS; **(C)** KCNN3; **(D)** KCNN4; **(E)** KCNMA1; **(F)** KCNJ2; **(G)** KCNA5.

As shown in Figure 6A, *NOS* positivity was reduced by 11 per cent units in littoral ECs (67% in the younger group and 56% in the older group) and 10 units in venous ECs (40% in the younger group and 30% in the older group). The frequency of *NOS3-*positive capillary ECs remained similar between the two groups, whereas lymphatic ECs expressing *NOS3* were 5 units higher in the old group.

In contrast to the overall eNOS downregulation, PTGIS frequency increased with age in arterial ECs (16 units), venous ECs (14 units), and particularly in VSMCs, which exhibited a 22-unit elevation. In contrast, *PTGIS* was low-abundant in capillary, littoral, lymphatic ECs, and pericytes from both age groups (Figure 6B).

Next, we examined the relationship between age and K+ channels (Figures 6C–G). Among genes typically expressed by ECs, *KCNN3, KCNN4,* and *KCNJ2* showed modest and variable differences between groups. Regarding mural cells, *KCNMA1* and *KCNA5* were 15 and 4 units higher in the VSMCs of the older group compared with the young group.

### Organotypic differences

**Figure 7:**
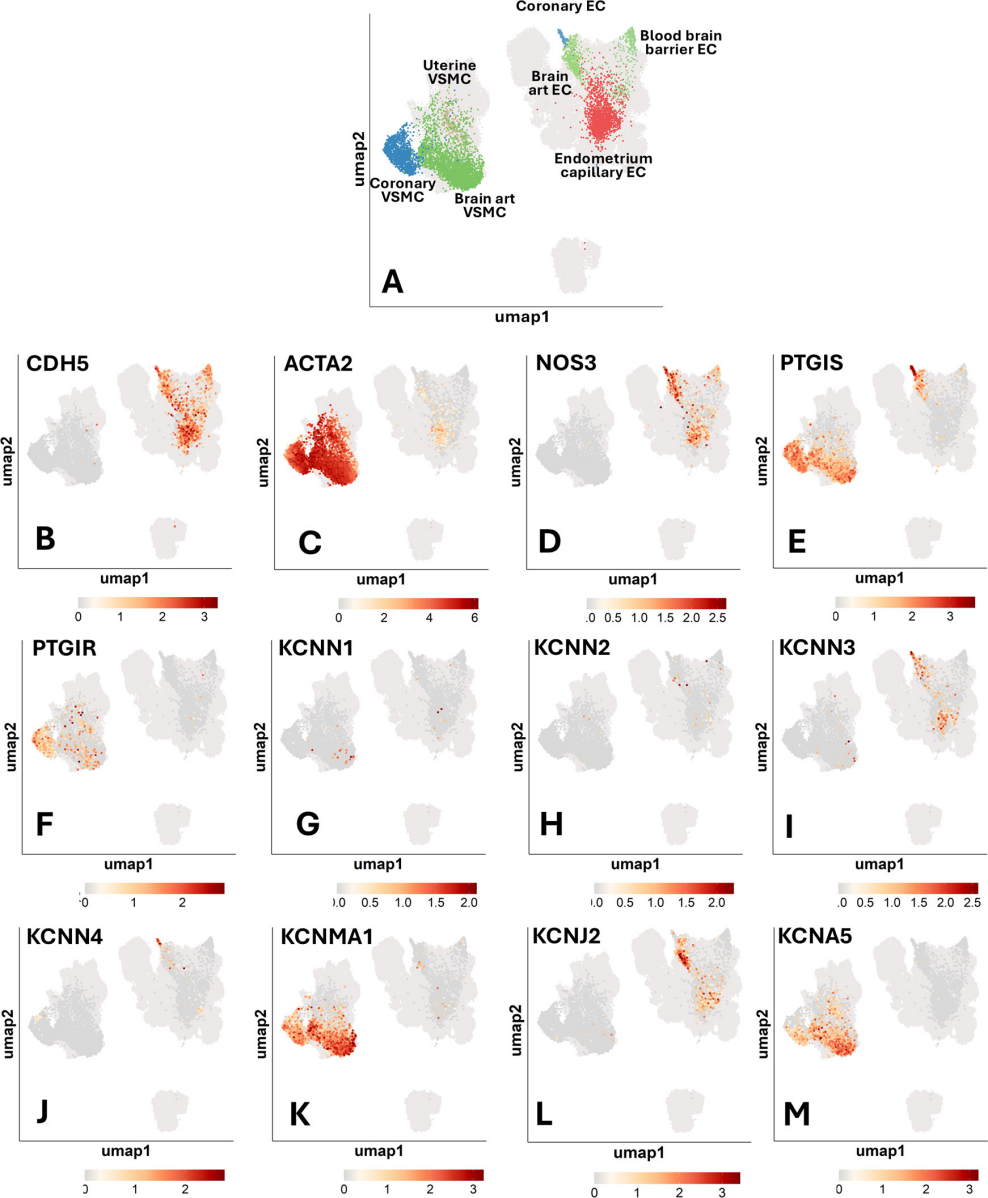
Single-cell transcriptomics of human vascular cells from the coronary arteries, brain, and uterus. UMAP representations of single-cell profiles and gene expression, side by side. The red scale indicates the level of gene expression. **(A)** UMAP representations of single-cell profiles. **(B)** CDH5; **(C)** ACTA2; **(D)** NOS3; **(E)** PTGIS; **(F)** PTGIR; **(G)** KCNN1; **(H)** KCNN2; **(I)** KCNN3; **(J)** KCNN4; **(K)** KCNMA1; **(L)** KCNJ2; **(M)** KCNA5.

**Figure 8:**
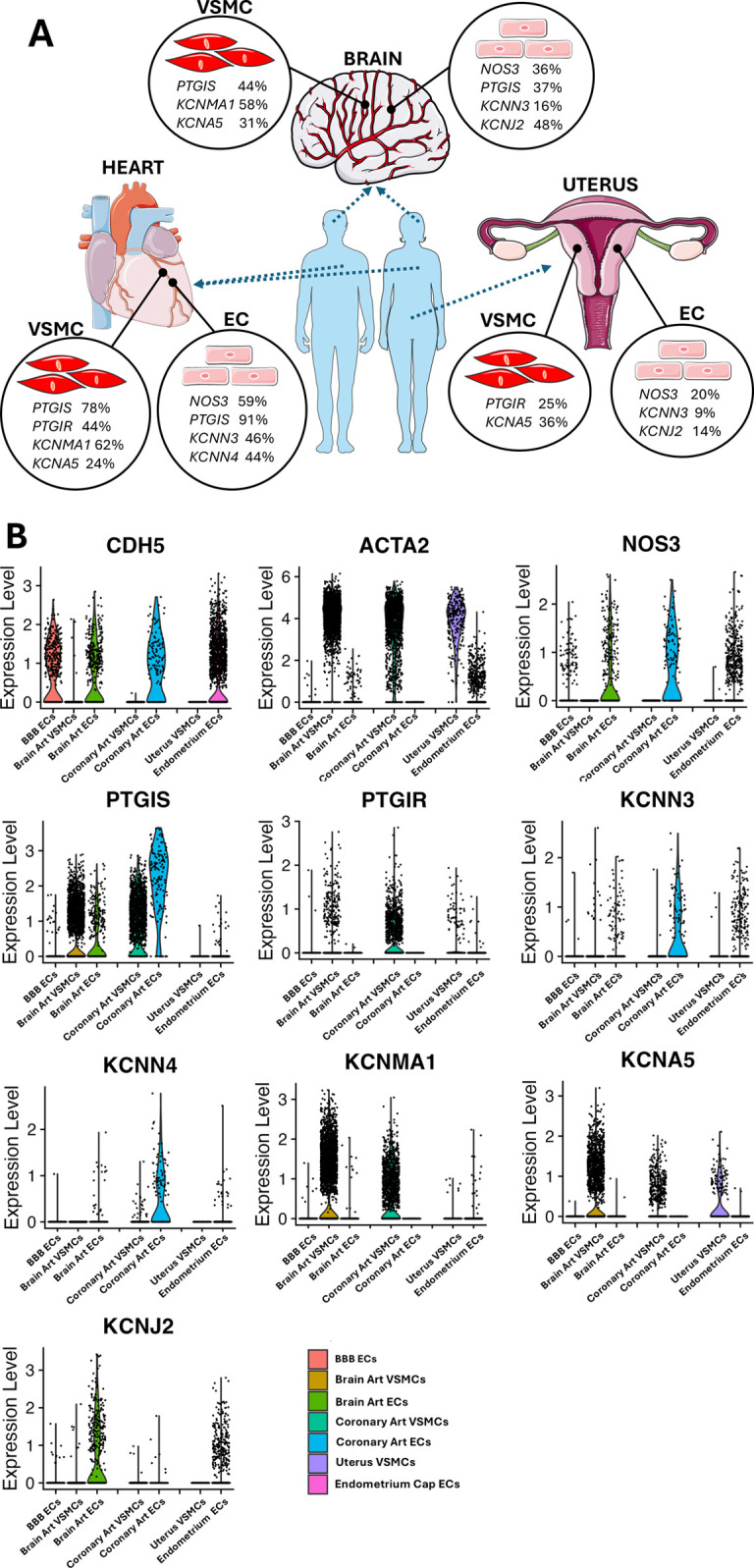
**(A)** Representative image highlighting the vascular gene transcripts that characterize each organ. Images from “Smart Servier Medical Art: Uterus”, “Heart”, “Brain circulation”, “Endothelium”, “Smooth muscle cell”, “Blue man shape” and “Human shape”, licensed under CC BY 4.0. **(B)** Coloured violin plot shape area represents the distribution of the expression within each condition tested. Several genes lack a coloured ‘violin’ shape and have instead a vertical line, which indicates that the overall expression of that particular gene is zero in most of the cells. Genes shown are in order: CDH5; ACTA2; NOS3; PTGIS; PTGIR; KCNN3; KCNN4; KCNMA1; KCNA5; KCNJ2.

Finally, we extracted and analyzed the data concerning ECs and VSMCs obtained from coronary arteries, the brain, and the uterus. Supplementary Table S3 reports the percentage of cells expressing a given gene. Figure 7 shows the UMAP representation of single-cell profiles. Figure 8 provides a violin plot representation of data distribution across different cells and organs.

*NOS3* was found to be expressed in ECs, with decreasing frequency in coronary ECs (59%), brain artery ECs (36%), blood-brain barrier (BBB) ECs (24%), and endometrium capillary ECs (20%) (Figure 7D). *PTGIS* exhibited a distinct expression frequency in arterial ECs and VSMCs from coronary arteries (91% and 78% positive cells, respectively) and brain (37% and 44% positive cells, respectively), whereas it was low expressed in BBB ECs (5% positive cells) and nearly absent in the endometrium capillary ECs and uterine VSMCs (Figure 7E). The PGI_2_ receptor gene *PTGIR* was exclusively expressed in VSMCs from coronary arteries (44%), uterus (25%), and brain (6%) and absent in ECs (Figure 6F). *KCNN3*, *KCNN4*, *KCNMA1*, *KCNJ2* and *KCNA5* emerged as the most predominant K+ channels, whereas *KCNN1* and *KCNN2* were expressed at very low levels (Figures 7G–M). In particular, *KCNN3* was present in all ECs, with the greatest frequency in coronary artery ECs (46%) followed by brain artery ECs (16%) and endometrium capillary ECs (9%). *KCNN4* exhibited a similar expression pattern, with higher values in coronary ECs (46%), followed by brain artery ECs (5%), and negligible levels in other tissues. *KCNMA1* and *KCNA5* were identified in the VSMCs of the coronary arteries (62% and 24%, respectively) and brain (58% and 31%, respectively). Moreover, *KCNA5* was detected in VSMCs from the uterus (35%), *KCNJ2* was mainly expressed in brain artery ECs (48%), followed by endometrium capillary ECs (14%), coronary ECs (5%) and BBB ECs (3%).

As shown in Figure 8, an organotypic specification is supported by the strikingly higher abundance of *NOS3* and *PTGIS* in coronary artery ECs (59% and 91% positive cells, respectively) as compared to the levels found in the global EC spectrum (27% and 24%, respectively). A similar enrichment was also observed regarding the K+ channels *KCNN3* and *KCNN4*, each being expressed in 46% of coronary artery ECs but only in 23% and 8% of the global ECs. Further evidence for an organotypic identity is provided by the data extracted from coronary artery and brain artery VSMCs. For instance, *PTGIS* was remarkably more frequent in coronary artery VSMCs (78%) and brain artery VSMCs (43%) as compared with global VSMCs (21%). A similar trend was observed when comparing the abundance of *PTGIR* transcripts in coronary artery VSMCs (44%) and global VSMCs (18%). A similar organ-specific enrichment was observed concerning *KCNMA1* for both coronary artery and brain artery VSMCs (62% and 57%, respectively vs. 37% in global VSMCs) and *KCNJ2* limited to coronary artery VSMCs (48% vs. 2% in global VSMCs). Although to a lesser extent, *KCNQ1* was expressed with higher frequency (between 10% and 15%) by brain vascular cells compared with the coronary artery and uterine vascular cells (Supplementary Table S3).

### Clustering analysis

**Figure 9:**
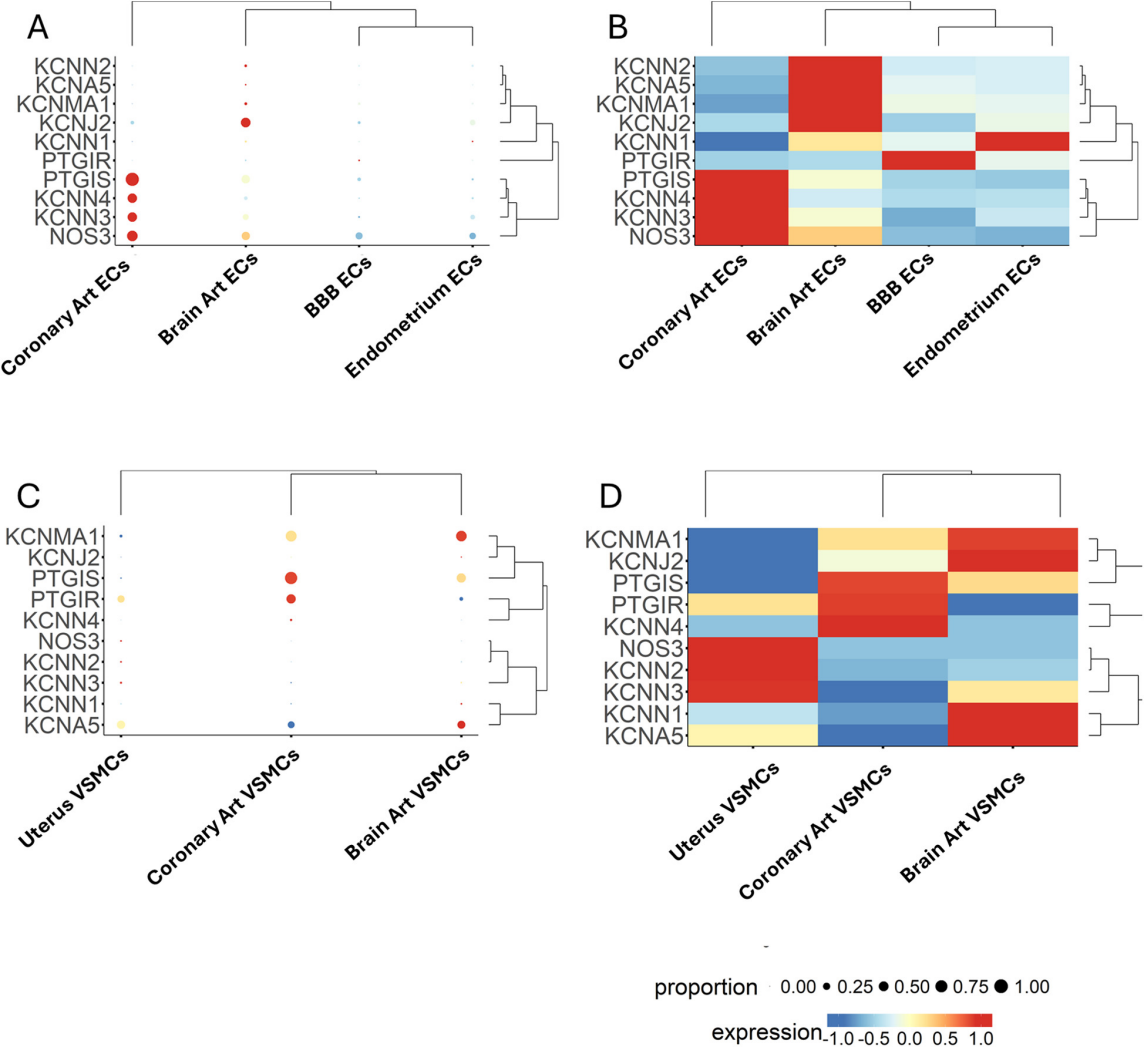
Expression patterns of multiple genes, grouped by categorical cell information, are displayed as either a bubble plot **(A, C)** or a heatmap **(B, D)**. Genes and cell populations are grouped into clusters, and dendrograms illustrate their relative distance. Bubble dimensions are proportional to the number of positive cells, while the heatmap colours identify the expression level as indicated by the scale.

As shown in Figures 9A,B, coronary artery ECs exhibited a distinct expression pattern compared to brain ECs, BBB ECs, and endometrium ECs. A remarkable difference was that coronary artery ECs were abundant in *NOS3* and *PTGIS* and showed a higher frequency and intensity for *KCNN3* and *KCNN4*. In contrast, brain ECs were characterized by a moderate abundance of *NOS3*, *PTGIS*, *KCNN3*, and *KCNJ2*, with the latter forming a cluster with a low-abundance, high-intensity gene set formed by *KCNN2*, *KCNA5*, and *KCNMA1*. BBB ECs showed higher *PTGIR* expression, although at low frequency levels. Endometrium capillary ECs exhibited moderate abundance of *NOS3*, *KCNN3*, and *KCNJ2*, along with the *KCNN1* gene expressed with high intensity but low frequency. Moreover, a considerable heterogeneity was identified when comparing VSMCs from the three districts (Figures 9C,D). Coronary artery VSMCs showed a cluster formed by *PTGIS* and *PTGIR*, together with *KCNN4,* which was, however, expressed only at low frequency. Brain artery VSMCs displayed two distinct clusters, namely *PTGIS, KCNMA1*, and *KCNJ2* (the latter being very low abundant but intensely expressed), and *KCNN1*, *KCNN3* and *KCNA5* (the first two exhibiting low abundance and intense expression, and the last one being both abundant and intensely expressed). Uterine VSMCs could be distinguished by a low-frequency/intense expression cluster formed by *NOS3*, *KCNN2*, and *KCNN3*. Moreover, they also showed intense and abundant expression levels regarding *KCNA5.*

## Discussion

This study examined single-cell transcriptomics of essential genes linked to vasorelaxation. Results indicate a considerable diversity at both the organismal and tissue levels, with age also contributing to variability.

## Angiotypic and organotypic diversity: can these concepts be harmonized?

Recent advancements in single-cell genomics and biological network analysis have significantly enhanced our understanding of the variety, flexibility, and modularity of vascular cells (26). These emerging technologies could also help to understand whether the transcriptional profile of vascular cells is tailored to specific organs and tissues or adheres to a more generalized angiotypic program. In a recent groundbreaking study, Barnett and colleagues reported that the global transcriptomic profile of a wide range of human vascular cells primarily corresponds to an angiotypic identity (20). These data contradict previous findings about mouse vascular endothelium, where an organotypic identity reportedly predominated (27). A targeted analysis of specific functions could help resolve the dispute. In this context, we examined the single-cell transcriptomics of key genes related to vasorelaxation in arterial and capillary ECs and arterial VSMCs at both the organismal- and tissue-specific levels. In addition, the expression of the same genes was analyzed in venous, lymphatic, and littoral ECs and pericytes.

## Expressional heterogeneity of redundant vasorelaxant pathways

Three separate yet interactive processes regulate vasorelaxation: the NO-cGMP pathway, the PGI_2_-cAMP pathway, and the hyperpolarization-K+ channels pathway. Cellular heterogeneity and modular redundancy collaborate in enhancing the functional flexibility and adaptability of the vascular system from large arteries to arterioles and capillaries (28). Accordingly, the proportional expression of the three pathways is anticipated to differ along the vascular tree. NO, a crucial signaling molecule, is synthesized by three NOS isoforms, all of which employ L-arginine and molecular oxygen as substrates (29). NOS1 is pivotal in central and peripheral neurons. Its roles encompass the regulation of synaptic plasticity, central control of blood pressure, and vasodilation through peripheral nitrergic neurons. Inducible NOS2 can be expressed in several cell types in response to lipopolysaccharide, cytokines, or other stimuli. It produces substantial amounts of NO, exerting cytostatic effects on parasite target cells. NOS3 is predominantly present in the endothelium. It maintains vasodilation, regulates blood pressure, and possesses several additional vasoprotective effects. We verified the dominant expression of *NOS3* in ECs identified by the co-expression of *PECAM1* and *EGFL7*. All other isoforms were not expressed (less than 1% of the cell fraction) except for *NOS1* in arterial ECs (7% positive cells) and *NOS2* in lymphatic ECS (3% positive cells) (data not shown). Furthermore, the results reveal a remarkable angiotypic variability in *NOS3* expression. Littoral ECs exhibited the most abundant expression of *NOS3* relative to other EC subtypes. This finding aligns with the fundamental roles of littoral ECs, which filter for antigens, pathogens, and senescent red blood cells, utilizing NO as a permeabilizing and cytotoxic agent (30, 31). The observed abundance of *NOS3* transcripts in arterial ECs compared with capillary ECs corresponds with prior findings indicating that this enzyme is predominantly confined to the endothelium of medium to large arterial blood arteries (21). A notable percentage of *PECAM1*- or *EGFL7*-positive ECs, especially those from the capillary region, were negative for *NOS3*. Additional research is required to ascertain the transcriptional repressor limiting *NOS3* expression, and to understand whether *NOS3*-negative cells can be transcriptionally recruited to augment NO synthesis in response to environmental cues, along with gene expression upregulation in constitutively *NOS3*-positive ECs.

The diverse properties of ECs were corroborated in relation to PTGIS, which had the highest frequency in arterial and venous ECs, while exhibiting low abundance in capillary, lymphatic, and littoral ECs. *PTGIS* enables to differentiate between VSMCs, positive for this enzyme, and pericytes, which are negative. *PTGIR* was exclusively expressed in mural cells, exhibiting comparable frequencies in VSMCs and pericytes. By engaging its corresponding receptors, PGI_2_ exerts cardiovascular actions, including vasodilation and the suppression of VSMC proliferation (32). Moreover, PGI_2_ is said to influence pericytes to preserve vascular barrier integrity and capillary perfusion (33).

Regarding endothelial K+ channels, we observed a decrease in *KCNN3* and *KCNJ2* from arterial to capillary ECs. At the same time, the muscular K+ channels *KCNMA1* and *KCNA5* exhibited a greater abundance in VSMCs than in capillary pericytes. These data are compatible with the major role of muscular arterioles in regulating vascular resistance, with capillaries playing a subsidiary contribution through the contractile activity of pericytes.

Co-expression and clustering analyses further validated the heterogeneity of arterial and capillary ECs. The data indicated that, within each EC subgroup, the most prevalent genes—*NOS3*, *PTGIS*, and *KCNN3*—were infrequently co-expressed, with over 50% of the cells exhibiting negativity for any of the examined genes. In resistance arteries, elevated endothelial Ca^2+^ levels facilitate vasodilation by activating SKCa/IKCa channels in certain vascular beds and stimulating eNOS in others (34). An interaction between the two pathways can amplify induced vasorelaxation. For instance, endothelial SKCa and IKCa channel activators have been demonstrated to augment agonist-induced membrane hyperpolarization, Ca^2+^ transients, and the *de novo* NO generation (34). Based on our findings on limited co-expression, it is plausible to hypothesize that the modularity of vasorelaxant processes is governed by the interaction among various ECs rather than within a distinct group of ECs co-expressing all three genes.

## Age-related decline in vasorelaxant gene expression

Increased age correlates with endothelial dysfunction. The possibility that hyperpolarization-dependent vasodilation may compensate for reduced NO generation is intriguing. However, studies in experimental animal models suggest that a diminished expression and activity of K+ channels could also contribute to the dysfunction of older blood vessels (35).

In our study, *NOS3* expression was consistently reduced in various EC types from the older cohort, while *PTGIS* frequency significantly escalated with age in arterial and venous ECs, and VSMCs. Similarly, *KCNJ2*, which encodes the Inward Rectifier Potassium Channel 2 and is regarded as a facilitator and maintainer of hyperpolarization, exhibited a significant increase in arterial ECs. The large-conductance KCNMA1 was almost twice as frequent in VSMCs of the older group compared to the younger group.

## Organotypic specification

The transcriptome analysis revealed similarities and disparities across vascular cells from the brain, heart, and uterus. *NOS3* exhibited a threefold greater frequency in coronary artery ECs than endometrium-derived ECs. Similarly, the brain and cardiac endothelium had a higher frequency of *PTGIS*, starkly contrasting ECs originating from the uterus. Likewise, significant variations in expression were noted with VSMCs. For instance, VSMCs from cerebral and cardiac arteries had high expression levels of *PTGIS*, whereas uterine VSMCs were devoid of this gene expression. The *PTGIR* gene was predominantly expressed in coronary and uterine VSMCs compared to cerebral VSMCs.

There is an increasing interest in the organotypic identity of K+ channels, mainly fuelled by the potential for targeted therapeutic applications. *KCNN3* was a common component across the three studied organs, whereas *KCNJ2* was found in cerebral and endometrium capillary ECs, albeit more extensively in the former. The big conductance BK channels, composed of pore-forming *α* subunits (BK-α, encoded by the *KCNMA1* gene) and regulatory β1 subunits (BK-β1, encoded by the *KCNMB1* gene), are prevalent in coronary arteries and modulate myocardial perfusion by linking intracellular Ca^2+^ homeostasis with excitation-contraction coupling (36–39). The voltage-dependent K+ (KV) subtype 5 channels, encoded by *KCNA5*, play a crucial role in regulating the precise coupling of coronary blood flow to myocardial metabolism (40). Our findings validated the elevated prevalence of *KCNMA1* and *KCNA5* gene transcripts in coronary artery VSMCs. This pattern was notably detected in brain VSMCs, but not in uterine VSMCs, which were abundant in *KCNA5* but displayed a low frequency of *KCNMA1*. Evidence suggests that K+ channels play a crucial role in modulating uterine vascular tone and responding to pregnancy and hypoxia (41). Alterations in the expression of these channels have been documented during pregnancy and are regarded as potential therapeutic targets for pregnancy-related disorders (42). Elevated NO production and augmented endothelium-derived hyperpolarizing factor-mediated responses, chiefly via the activation of small and intermediate conductance K+ channels, significantly contribute to endothelium-dependent vasorelaxation of the uterine artery in nonpregnant rats, rising to approximately 70% in pregnant rats (43). Our observations regarding the abundant expression of *KCNA5*, which encodes the Voltage-Gated Potassium Channel Protein Kv1.5, suggest this channel may have a physiologic role in regulating uterine circulation. Seminal research in non-pregnant sheep showed that the administration of 4-aminopyridine (4-AP), a selective Kv channel blocker, did not affect the baseline uterine blood flow but diminished the estradiol-induced increase in uterine blood flow (44). Dysfunction of Kv channels may contribute to pregnancy complications, as indicated by findings showing hypoxia specifically diminished Kv currents assessed via the patch clamp technique in VSMCs from fetoplacental arteries and triggered vasoconstriction in placental cotyledons (44).

## Comparison with global levels confirms organotypic identity

An additional insight favoring organotypic identity is provided by the large enrichment of several genes in ECs and VSMCs from coronary and brain arteries compared with organismal expression levels. For instance, we observed a striking enrichment in NOS3, PTGIS, and PTGIR in coronary artery vascular cells, accompanied by a higher abundance of K+ channels, specifically *KCNN3*, *KCNN4*, *KCNMA1*, and *KCNJ2*. These data are consistent with the potent vasorelaxant capacity of coronary arteries through multiple, redundant mechanisms. A similar, though lesser enrichment was observed in vascular cells from the brain circulation.

## Therapeutic perspectives

There is a great interest in the possibility that single-cell transcriptomics would provide a means to refine personalized treatments. Based on patients' specific requirements, one can opt for a generalized strategy addressing all primary vasorelaxant mechanisms or focus on certain vascular beds informed by identifying angiotypic and organotypic variability. Given its preliminary nature, we are far from claiming that this study can guide a personalized approach. However, in this section, we would like to present how a better understanding of vascular cells' heterogeneity may provide an alternative to complex multidrug treatments. The recent Lacunar Intervention Trial-2 (LACI-2) shows a global methodology (45). This interventional trial aimed to administer increasing dosages of isosorbide mononitrate, a NO donor, and Cilostazol, a phosphodiesterase type 3 inhibitor that enhances the prostacyclin-cAMP pathway in stroke patients. The findings demonstrate that the treatment diminished the risk of significant vascular incidents and improved functional and cognitive results relative to single-agent therapy, while maintaining a favorable safety profile (45). Rinvecalinase alpha (DM199) is a recombinant variant of the endogenous protein tissue kallikrein, presently undergoing trials in patients with strokes and complicated pregnancies (46). It is a bioactive kinin peptide source that concurrently activates the three principal vasorelaxant systems (47). On the other hand, K+ channel modulators offer a novel and appealing approach to target specific arterial beds selectively. Endothelial SKCa and IKCa activators may be appropriate for treating vascular problems in the brain, heart, and uteroplacental unit. Small-molecule activators of KCa2.x and 3.1 channels, including SKA-31, can rapidly suppress myogenic tone in isolated resistance arteries, promote significant vasodilation in intact vascular systems like the coronary circulation, and promptly reduce systemic blood pressure *in vivo* (48). At the same time, BKCa agonists may be effective for targeting the brain or heart specifically. Several clinical trials using BKCa openers for cardiovascular conditions, including hypertension, ischemic heart disease, stroke, and erectile dysfunction, have been commenced in recent decades. Nevertheless, the inadequate potency and lack of selectivity of these compounds led to the premature cessation of these trials (49). Only one medication candidate, andolast, targeting BKCa channels, remains in clinical development for the treatment of asthma (50). Voltage-sensitive Transcriptional regulators are also appealing. The nuclear factor erythroid 2-related factor 2 (Nrf2) has been shown to upregulate BKCa expression by directly binding to the antioxidant response element (ARE) motif of the *KCNMA1* promoter, suggesting that treatment with FDA-approved Nrf2 activators may be an effective strategy for enhancing the treatment of coronary artery disease (38). Finally, activators of Kv channels could provide a means to induce extended vasorelaxation. We have demonstrated that VSMCs from coronary, cerebral, and uterine arteries express KCNA5, which encodes the Kv1.5 potassium channel. Recent studies have proposed Kv7 channels, encoded by *KCNQ 1–5* genes, as promising new therapeutic targets to increase fetoplacental blood flow in preeclampsia (51). Nonetheless, this gene family was lowly expressed in VSMCs from our series, except *KCNQ1,* which was found to be expressed by more than 10% of brain vascular cells.

## Conclusions and study limitations

Our data reveal considerable heterogeneity in the expression of vasorelaxant pathway components among vascular cells throughout the arterial tree and different organs. K+ channels and the PGI_2_-generating enzyme are posited to gain prominence in the ageing vasculature, perhaps offsetting the reduced expression of *NOS3*. K+ channels are a vital therapeutic target to improve the effectiveness of existing treatments utilizing NO donors and PGI_2_ analogues.

The study has several limitations intrinsic to its explorative nature. We analyzed a few “sentinels” of relaxation, which may represent a simplification of the complex transcriptomic scenario. This initial attempt justifies additional research to discern the complexity through the inclusion of other vasorelaxant mediators. Similarly, it is premature to infer the physiological implications of these novel data within the enormous body of knowledge on human vascular tissue and bulk cell preparations. A comprehensive discussion on functional repercussions should be deferred until additional data on vascular cells from many human subjects under physiological and pathological settings are available. This will allow for the understanding of how the modification of gene expression through the recruitment or derecruitment of single cell populations for a specific gene correlates with functional changes. Additional restrictions arise from the source database's cell count, which impeded the incorporation of covariates. Rather than examining expression variations across age percentiles, we analyzed two age groups below and above 50. More ageing research will be attainable as the Atlas data is anticipated to be expanded with additional contributions. Another limitation of single-cell data is that the low overall depth of sequencing per cell means that several “drop-out” genes, which are expressed in cells albeit at low levels, are not apparent in the final data. Single-cell sequencing has lower read depth and capture efficiency compared to bulk RNA sequencing or conventional PCR, making it harder to detect lowly expressed genes (52). Deepening the search from general cell populations into cells from specific organs may rule out the possibility of missing relevant gene transcripts. This was the case of the Kv7 channels, a type of voltage-gated K+ channel that plays a crucial role in regulating neuronal excitability and vascular cells' control of blood flow. Their expression in brain vascular smooth muscle cells and endothelium contributes to regulating vessel tone and maintaining the blood-brain barrier (53, 54). Similarly, Kv7 channels have been detected in coronary arteries of animal models and human chorionic plate arteries (51, 55). An analysis of the general cell population indicated that Kv7 gene transcripts were lowly expressed. However, when looking at specific organs, we found an enrichment of the encoding gene transcripts in brain vascular cells at least for the *KCNQ1* gene, while remaining low for *KCNQ 2–5*.

Finally, transcriptome data should be supplemented and validated by evaluating protein levels, potentially integrated with topographic analysis as shown in spatial transcriptomics and proteomics, and epigenetic mechanisms. Despite these limitations, our work lays the groundwork for formulating innovative hypotheses regarding vascular cell heterogeneity, which could potentially benefit patients. The most intriguing inquiries for future investigation involve the requisite number of additional vascular cells necessary to express the examined gene to observe alterations in vasorelaxation, and whether this objective can be therapeutically attained through transcriptional inducers, either independently or in conjunction with traditional activating ligands.

## Data Availability

The datasets presented in this study can be found in online repositories. The names of the repository/repositories and accession number(s) can be found in the article/Supplementary Material.
