# Supplementary material for: Single-cell transcriptomic analysis of the human vascular atlas provides new insights into vasorelaxation redundancy and heterogeneity
Source: Front Cardiovasc Med. 2025 Aug 13;12:1634645. doi: 10.3389/fcvm.2025.1634645 (PMC12381952; doi:10.3389/fcvm.2025.1634645)
Supplement: Supplementary file 1 [file Datasheet1.pdf]

Supplementary Table 1. Number of single cells screened and percentage of positive cells for typical endothelial and mesenchymal cell markers in each subpopulation.

Group	Total Cell N	*CDH5*	*PECAM*	*EGFL7*	*VWF*	*ACTA2*	*PDGFRB*	*NOTCH3*	*MYH11*	*RGS5*
**Arterial EC**	5153	68.54	90.51	81.10	70.00	4.08	1.46	0.93	3.40	5.82
**Capillary EC**	23192	67.01	81.67	85.58	74.26	7.61	1.88	1.81	2.77	15.50
**Venous EC**	6088	64.14	95.40	77.12	96.88	4.14	1.03	0.92	9.41	12.50
**Littoral EC**	4845	26.93	73.23	74.12	65.47	0.53	0.23	0.31	1.01	0.43
**Lymphatic EC**	4232	47.71	76.18	74.88	43.71	3.97	0.80	0.40	1.13	1.70
**VSMC**	16727	0.21	0.57	0.82	1.11	98.43	59.37	72.36	94.00	60.76
**Pericytes**	6386	1.28	1.89	5.47	3.68	82.76	78.32	73.74	24.32	91.97

Supplementary Table 2. Number of single cells screened and percentage of positive cells for the indicated genes.

Group	Total Cell N	*NOS3*	*PTGIS*	*PTGIR*	*KCNN1*	*KCNN2*	*KCNN3*	*KCNN4*	*KCNMA1*	*KCNJ2*	*KCNA5*	*KCNQ1*	*KCNQ2*	*KCNQ3*	*KCNQ4*	*KCNQ5*
**Arterial EC**	5153	26.76	24.72	0.21	0.21	2.15	23.13	7.94	0.56	18.65	0.17	3.03	1.40	0.70	0.33	0.02
**Capillary EC**	23192	15.66	3.45	0.26	0.22	2.59	5.19	0.41	0.25	3.10	2.15	3.91	0.78	0.21	0.07	0.02
**Venous EC**	6088	32.8	27.35	0.08	0.31	1.72	11.6	2.15	0.21	1.38	0.05	2.30	0.05	0.67	0.16	0
**Littoral EC**	4845	61.84	0.08	0.19	0	1.84	16.84	0.23	5.35	2.89	0.02	0.54	0.14	3.03	0	0.02
**Lymphatic EC**	4232	15.88	2.63	0.05	0.07	2.32	14.77	0.19	0.38	3.47	0.00	0.78	0.09	0.69	0.02	0.05
**VSMC**	16727	0.07	21.84	18.22	0.34	0.07	0.32	0.24	37.8	2.16	31.63	3.75	0.07	2.68	3.66	0.81
**Pericytes**	6386	0.34	0.83	17.24	0.19	0.05	0.24	0.08	0.78	0.30	1.99	0.09	0.09	1.63	1.25	0.13

Supplementary Table 3. Number of single cells screened and **p**ercentage of positive cells for the indicated genes, relative to the organs.

Group	Total Cell N	*CDH5*	*ACTA2*	*NOS3*	*PTGIS*	*PTGIR*	*KCNN1*	*KCNN2*	*KCNN3*	*KCNN4*	*KCNMA1*	*KCNJ2*	*KCNA5*	*KCNQ1*	*KCNQ2*	*KCNQ3*	*KCNQ4*	*KCNQ5*
**Coronary Art ECs**	138	68.84	0	58.70	91.30	0	0	0	46.38	46.38	0	5.07	0	0	0	1.45	1.45	0
**Coronary Art VSMCs**	1461	0.07	96.58	0	78.17	43.60	0.14	0	0.14	1.71	62.08	48.36	23.55	0	0.07	1.44	4.72	4.86
**Blood brain barrier ECs**	368	67.12	2.17	24.18	5.16	0.82	0.82	0.54	1.09	0.27	1.93	2.99	0.27	14.13	1.09	0.82	0.54	0
**Brain Art ECs**	426	53.29	12.91	35.68	36.62	0.47	1.41	2.35	16.24	5.16	3.05	14.36	0.47	11.03	0.47	1.88	0.23	0
**Brain Art VSMCs**	3260	0.15	99.11	0	43.59	6.01	0.64	0.06	0.52	0	57.85	0.27	30.98	10.03	0	6.66	3.53	0.43
**Endometrium Cap ECs**	1755	57.55	16.81	20.17	1.24	0.57	0.51	1.65	9.23	1.37	1.08	0.25	0.11	6.32	0.34	1.31	0.11	0
**Uterus VSMCs**	252	0	98.81	0.79	0.41	25.12	0.43	0.79	1.19	0	2.78	0	35.71	0	0	0	6.35	0
